# Sociodemographic, Clinical Variables, and Quality of Life in Patients with Epilepsy in Mekelle City, Northern Ethiopia

**DOI:** 10.1155/2018/7593573

**Published:** 2018-12-02

**Authors:** Abadi Kahsu Gebre, Amdemicheal Haylay

**Affiliations:** Department of Pharmacology and Toxicology, School of Pharmacy, Mekelle University, Ethiopia

## Abstract

**Background:**

Epilepsy is a chronic neurological disorder characterized by unprovoked recurrent seizure episodes. The disease has detrimental effects on social, cognitive, psychological, and physical components of life consequently quality of life of the patients. The level of the effect of the disease on quality life is influenced by different factors including the use of antiepileptic medications.

**Objectives:**

The study was aimed at assessing quality of life in patients with epilepsy and the variables affecting it in Mekelle city, northern Ethiopia.

**Methods:**

175 patients with epilepsy aging 18 years old and above attending neurologic clinics of the two governmental hospitals available in Mekelle city were interviewed using standard and validated Tigrigna version of Quality of Life in Epilepsy Scale-31 (QOLIE-31). One-way ANOVA and independent *t*-test and analysis of covariance were used for data analysis.

**Result:**

The mean age of the patients was 29.36 (standard deviation (SD) 12.77) years old, and 61% of them were males while 52% of the respondents were on phenobarbitone monotherapy. The mean total QOLIE-31 score was 77.97 (SD 20.78) with the highest subscale score for medication effects and the lowest for overall quality of life (QOL) functioning with a score of 86.2 (SD 22.12) and 70.97 (SD 26.43), respectively. The patients with high seizure frequency in the past month before the current visit had a significantly low quality of life 76.81 (SD 21.11). Conversely, patients with tertiary education and above had shown a significantly high quality of life 89.52 (SD 11.85).

**Conclusion:**

The overall QOL of the patients was good. Seizure frequency and level of education were found significant predictors of QOL showing the necessity of seizure control and patient education for improving quality of life in patients with epilepsy.

## 1. Background

Epilepsy is a chronic neurological disorder characterized by recurrent episodes of unprovoked seizures with or without loss of consciousness [1]. Around 50 million people worldwide have epilepsy with 2.4 million of new cases appearing each year making it the most common neurological disorder. The disease is reported to have wide global distribution with high prevalence in the developing world such as in sub-Saharan African countries accounting for 80% of the prevalence [2].

In Ethiopia, although there is an obvious lack of credible and dependable epidemiologic data in the literature as in many of the occasions, review of community-based epidemiological studies of neurological disorders showed that epilepsy is a prevalent neurological disorder. The prevalence of the disease in the country was reported to be 5.2/1000 inhabitants at risk with the annual incidence of 64 in 100,000 inhabitants [3, 4].

Epilepsy is a worldwide public health concern often accompanied by physical and cognitive disability leading to limitation in employment, independence, and social activities [5]. Patients with epilepsy have greater difficulties dealing with schooling and are widely stigmatized [6]. These encounters may therefore affect the lifestyle and quality of life of the patients [7, 8]. In addition, shortness of medical services, unavailability of antiepileptic medications, and lack of awareness of medical treatment and cultural-related factors are more common phenomena in developing countries including Ethiopia [9]. These factors may also contribute to low quality of life among patients with epilepsy in Ethiopia. However, there are no studies conducted so far about health-related quality of life and antiepileptic medication use among patients with epilepsy in Ethiopia.

## 2. Methods

### 2.1. Study Area and Design

A cross-sectional study design was employed, and the data were collected by trained health care professionals from January to March, 2018 using QOLIE-31 questionnaire among patients with epilepsy visiting neurologic clinics of two governmental hospitals found in Mekelle city (Ayder Comprehensive Specialized Hospital (ACSH) and Mekelle Hospital) located in the northern part of Ethiopia, Tigray region, 783 km far from Addis Ababa. The questionnaire was translated into a local language, Tigrigna, and back translated into English to maintain its consistency by independent language experts.

### 2.2. Sampling Size Determination and Sampling Procedure

Sample size was determined using a single proportional formula considering 50% of prevalence as there was no related study in the area. The sample size was found to be 384 by taking 5% of margin of error at 95% confidence interval. As the patient flow was generally low in the hospitals, the patterns of patient flow to the neurologic clinics were also considered. The average patient flow rate per week was 30. The average patient visit within 4 months of data collection period was found 480. Out of these, the patients in every third interval were randomly selected. Adding 10% allowance to compensate for nonrespondents and incompleteness, 175 patients with epilepsy were included in this study.

### 2.3. Source Population

The source populations were all patients with epilepsy attending the neurologic clinic of ACSH and Mekelle Hospital during the study period.

### 2.4. Target Population

The target populations of the study were all patients with epilepsy who were on antiepileptic medications and who had at least one follow-up before the recent visit to the clinic.

### 2.5. Inclusion Criteria

Patients with epilepsy aging 18 years and above who were on antiepileptic medication at least for six months were included in this study.

### 2.6. Data Quality Control

Completeness of the data collected was supervised and monitored daily during the data collection processes.

### 2.7. Data Analysis

Data entry and data analysis were carried out using SPSS version 20 software. Descriptive statistics such as frequency, percentage, mean and standard deviation, ANOVA, and independent sample *t*-test were employed to compare means of QOL scores between groups and for the variables which showed a significant difference with ANOVA, frequency seizure, and level of education; correlation coefficient was used to measure the relationship with a total score of QOLIE-31.

### 2.8. Ethical Considerations

Ethical clearance was obtained from the ethical review board of the College of Health Sciences, Mekelle University, and permission for data collection was obtained from the Ayder Comprehensive Specialized Hospital management. Informed consent was also obtained for each patient prior to the data collection.

### 2.9. Operational Definition

Quality of life is the physical, psychological, and social well-being of patients with epilepsy.

Antiepileptic medications are drugs used to control epileptic seizure.

Adverse drug reaction (ADR) is an unwanted and undesirable effect of a medication that occurs during usual therapeutic use.

## 3. Result

### 3.1. Sociodemographic Characteristics of the Patients

Out of 175 patients with epilepsy included in this study, 107 (61.1%) of them were men while 68 (39.9%) were female. The mean ages of the patients were 29.36 ± 12.77. 100 (57.1%), 163 (93%), and 108 (61.7%) of the patients were urban dwellers, followers of Orthodox Christianity, and with a monthly income of less than 20 USD, respectively (Table 1).

The mean (SD) total score of QOLIE-31 was 77.97 ± 20.78. The highest mean score was for medication effect subscale (86.20 ± 22.12), while the lowest mean score was for overall QOL functioning subscale (81.68 ± 27.03) (Table 2).

One-way ANOVA and independent sample *t*-test evaluated the interrelation between QOLIE-31 scores, sociodemographic, and clinical parameters. Gender, age, education level, seizure frequency, antiepileptic medication, and adverse events of antiepileptic medication were variables studied in this sample. One-way ANOVA followed by post hoc analysis has shown a significant difference in mean total score of QOLIE-31 with level of education. Patients with no seizure episode during the last month had better mean total score of QOLIE-31 (91.28) as compared to the study participant having seizure in the last one month (76.81, *p* ≤ 0.001). Study participants with no formal education and primary education had the lowest mean total score QOLIE-31, 69.48 and 73.13, respectively (*p* value ≤ 0.001) as compared to individuals with tertiary and higher level of education (89.52) (Table 3).

The correlation between total score and QOLIE-31 subscales is described. Total quality of life showed a significant moderate negative correlation with number of seizures. Generally, increase in frequency of seizure decreases total quality of life score. Increase in seizure frequency primarily lowers seizure worry, emotional wellbeing, and energy/fatigue aspects of quality of life as has been shown by significantly moderate correlation. However, seizure frequency was found to have an insignificant effect on the other subscales of QOLIE-31 (Table 4).

Good level of education shows a positive correlation with total quality of life score. Increase in level of education has been associated with increase in quality of life of the patients. All of the quality of life subscales have shown a significantly moderate positive correlation with exception of medication effect which showed weak correlation (Table 5).

Most of the study participants were on phenobarbitone monotherapy (52%) followed by combination therapy of phenobarbitone and valproate and combination therapy of phenobarbitone and carbamazepine. The total QOLIE-31 score was high among carbamazepine users (Table 6).

Around three-fourth (73%) of the study participant did not experience adverse drug reaction or discomfort from AEDs, and 27% (48) of the study participant experience ADR; out of the 27% ADR, day time sedation was the predominant ADR; 17.7% and majority of the sedation was due to phenobarbitone monotherapy. Gingival hypertrophy was high with phenytoin (40%) (Table 7).

## 4. Discussion

Epilepsy is a chronic neurological disorder characterized by recurrent episode of unprovoked seizures with or without loss of consciousness [10]. Epilepsy affects many aspects of quality of life including social functioning, the ability to concentrate on things, the ability to work with full potential, psychological aspect, and other physical activity [1]. This study assessed the quality of life among patients with epilepsy who were on antiepileptic medication use. The mean total score QOLIE-31 of this study was 77.97 ± 20.78. This is higher than the study conducted in Malaysia (mean score of 68.9 ± 15.9) [11], Australia (mean score of 52.9 ± 23.1) [12], Moscow (42.12 ± 4.14) [13], Benin (52.1 ± 33.4) and Togo (49.5 ± 14.4) [14], and India (64.61 ± 8.97) [15], but it is lower than the study conducted in Canada (82 ± 32.8) [16]. The relatively better mean total score of QOLIE-31 in this study may be due to better medical care as patients with epilepsy are treated by neurologists in both hospitals. It could also be due to the difference in the way of translating the questionnaire, difference in methodology, study setting, and inclusion criteria as patients taking antiepileptic medication six months and above were included in our study. It could also be due to the sociocultural difference of the study participants as cross-cultural differences were found to influence QOL in patients with epilepsy [17].

In this study, medication effect had the highest mean score, 86.20 ± 22.12. This indicates the patients have experienced low mental and physical effects related to AEDs. In contrast, overall QOL functioning was with the lowest mean score, 70.97 ± 26.43, which indicates things in the past time were worse and difficult. Medication effect subscale score of QOLIE-31 of this study is similar with the studies conducted in West Africa [14] and Malaysia [11], where medication effects had the highest score. However, it is not similar with the lowest score in which seizure worry had the lowest score in these studies. This difference is due to variation in psychological, sociocultural belief, and life style of the patients.

Level of education is sociodemographic parameter that has shown a significant variation in means score of QOLIE-31 in this study. This is similar with the study conducted in Indonesia [18], South Korea [19], and Georgia [20], whereby level of education was one of the predictor of quality of life in patients with epilepsy. This could be due to the influence of education on individual perception of their disease condition and adherence to their medications.

The study conducted in Georgia shows that being female sex was associated with low seizure worry score which indicates that being female was a risk for worry and fear for seizure. Conversely, sex was not related with poor quality of life in this study [20].

In this study, there were no significant association between type of antiepileptic medication and total QOLIE-31 score. This could be due to comparable efficacy and safety of the medications. Almost half (52%) of the study participant were on phenobarbitone monotherapy. This may be due to the affordable cost and better availability of the drug. This is in agreement with a study conducted in Estonia [21].

Seizure frequency was a stronger predictor of poor quality of life, because it is associated with excess fear, unable to work, stigmatization, diminishing hope and future life, impairment in social function, and psychological impairment [22]. This study was similar with the studies that were conducted in Mexico [23], Georgia [20], Malaysia [15], Benin and Togo [17], and UK [24], in which frequent seizure episode was associated with poor quality of life than few or no seizure episode. Individuals with frequent epileptic seizures will always be in discomfort, and they do not know when the next seizure will happen, as a result they take care and impose restrictions from driving, cooking, and doing risky jobs to avoid having seizure episodes at inappropriate times, places, or social events. These in turn may compromise the quality of life of the patients.

## 5. Conclusion

The quality of life of patients attending in the hospitals is good. Frequent seizure and level of education was the predictor of poor quality of life among patients with epilepsy.

## 6. Limitation

This study was conducted in governmental tertiary hospitals whereby the follow-up is carried out by experienced neurologist's specialists. Therefore, the score may not reflect the quality of life of patients with epilepsy in other health care system.

## Figures and Tables

**Table 1:** Sociodemographic characteristics of respondents.

Sociodemographic parameter	*N* (%)
Sex	
Female	68 (38.9)
Male	107 (61.1)
Residence	
Urban	100 (57.1)
Rural	75 (42.7)
Religion	
Orthodox	163 (93.1)
Muslim	10 (5.7)
Catholic	1 (0.6)
Protestant	1 (0.6)
Marital status	
Single	97 (55.4)
Married	69 (39.4)
Divorced	6 (3.4)
Widowed	3 (1.7)
Level of education	
No formal education	26 (14.9)
Primary	47 (26.9)
Secondary	72 (41.1)
Tertiary	30 (17.1)
Monthly income	
<20$	108 (61.7)
20 up to 60$	22 (12.6)
>60$	45 (25.7)

*N* = number of patients.

**Table 2:** QOLIE-31 subscale score among patients with epilepsy in ACSH and Mekelle Hospital.

Subscale of QOLIE-31	Mean (SD)
Seizure worry	82.17 (22.76)
Over all QOL	70.97 (26.43)
Emotional wellbeing	73.83 (22.35)
Energy/fatigue	75.25 (17.88)
Cognitive	80.06 (24.24)
Medication effect	86.20 (22.12)
Social function	81.68 (27.03)
Total score	77.97 (20.78)

SD = standard deviation; QOLIE = quality of life in epilepsy. Independent sample *t*-test was performed to show the significance of sociodemographic parameter, drug therapy, presence or absence of seizure, and adverse drug reaction on mean total QOL score of QOLIE-31.

**Table 3:** Difference of QOL score in epilepsy patient in ACSH and Mekelle Hospital.

Parameter	*N* (%)	Total QOL score (Mean (SD))	*P*
Sex			
Female	68 (38.9)	80.21 (20.37)	0.888^a^
Male	107 (61.1)	76.55 (21.01)	
Residence			
Urban	100 (57.1)	78.33 (21.72)	0.245^a^
Rural	75 (42.9)	77.5 (19.59)	
Level of education^c^			
No formal education^d^	26 (14.9)	69.48 (21.27)	0.001^b^
Primary^a^	47 (26.9) 72 (41.1)	73.13 (21.88)	
Secondary	30 (17.1)	79.38 (20.76)	
Tertiary and above		89.52 (11.85)	
ADR			
Yes	48 (27.4)	72.81 (22.14)	0.273^a^
No	127 (72.6)	79.92 (19.98)	
Presence of seizure per month^c^			
Absence	14 (8)	91.28 (9.21)	0.001^a^
Present	161 (92)	76.81 (21.11)	
Drug therapy			
Monotherapy	121 (69.1)	79.58 (20.24)	0.785^a^
Polytherapy	54 (30.1)	74.37 (21.7)	

^a^Independent sample *t*-test; ^b^one-way ANOVA; ^c^significance consider when *p* < 0.05; SD = standard deviation; *N* = number of patients; ADR = adverse drug reaction; QOL = quality of life; ^d^*p* value ≤ 0.001; ^e^*p* value 0.003.

**Table 4:** Correlation between number of seizure and total score of QOLIE-31 subscale in ACSH and Mekelle Hospital.

Subscales of QOLIE-31	Correlation coefficient	*p* value
Seizure worry	−0.152	0.045
Over all QOL	−0.138	0.069
Emotional wellbeing	−0.160	0.035
Energy/fatigue	−0.200	0.008
Cognitive functioning	−0.133	0.080
Medication	−0.123	0.104
Social functioning	−0.141	0.063
Total score	−0.168	0.026

**Table 5:** Correlation between level of education and total score of QOLIE-31 subscale in ACSH and Mekelle Hospital.

Subscales of QOLIE-31	Correlation coefficient	*p* value
Seizure worry	0.169	0.026
Over all QOL	0.224	0.003
Emotional wellbeing	0.208	0.006
Energy/fatigue	0.303	0.001
Cognitive functioning	0.307	0.001
Medication effect	0.147	0.052
Social functioning	0.285	0.001
Total score	0.299	0.001

**Table 6:** Mean total QOL score and antiepileptic medications use by the research participants in ACSH and Mekelle hospital.

Type of medication	*N* (%)	Mean (SD)^b^
Phenobarbitone	91 (52)	77.89 (20.68)
Carbamazepine	15 (8.6)	83.54 (20.55)
Phenobarbitone and valproate	23 (13.1)	71.12 (19.99)
Phenobarbitone and carbamazepine	19 (10.9)	75.09 (24.06)
Others	27 (15.4)	83 (18.89)
Total^a^	175 (100)	77.97 (20.78)

^a^Phenytoin, valproate, combination of phenytoin and valproate, and combination of CZP and phenytoin; ^b^*p* value < 0.238 (not significant).

**Table 7:** Reported adverse drug events of AEDs.

Responsible drug		Adverse drug reaction				
	N	Sedation	Gingival hypertrophy	Diplopia	GI discomfort	Dizziness
Phenobarbitone	91	14		1	2	—
Carbamazepine	15	1	—	—	1	2
Phenobarbitone and valproate	23	8	—	—	—	1
Phenobarbitone and carbamazepine	19	1	—	—	2	1
Valproate	8	—	—	1	1	—
Phenytoin	8	1	2	—	—	—
Others^a^	10	5	3	—	—	—
Total	175	31	5	2	6	4

Others include: phenobarbitone, carbamazepine, and valproate; phenobarbitone, carbamazepine, and phenytoin; phenytoin and valproate. ^a^Only patients with phenytoin containing regimen experienced gingival hypertrophy.

## Data Availability

The data used to support the findings of this study are included within the article.
